# Trends in immunomodulatory therapy for the treatment of chronic uveitis in the United States

**DOI:** 10.1186/s12348-025-00552-z

**Published:** 2025-11-25

**Authors:** Charles Zhang, Sinan Ersan, Jonathan B. Lin, Mario Cale, Thomas A. Albini

**Affiliations:** 1 Department of Ophthalmology, Bascom Palmer Eye Institute, University of Miami Miller School of Medicine, 900 NW 17th Street, Miami, FL 33136 USA; 2 Department of Ophthalmology, Ross Eye Institute, SUNY Buffalo, Buffalo, NY USA; 3 Jacobs School of Medicine and Biomedical Sciences, University at Buffalo, Buffalo, NY USA; 4 Department of Ophthalmology, Byers Eye Institute, Stanford University School of Medicine, Stanford, CA USA

**Keywords:** Chronic uveitis, Immunomodulatory therapy, Methotrexate, Adalimumab, Medical treatment

## Abstract

**Background:**

Chronic uveitis, a major cause of legal blindness in the United States, often requires long-term immunomodulatory therapy (IMT). This study aims to characterize trends in systemic IMT use among adults with chronic uveitis in United States from 2005 to 2025.

**Methods:**

This cross-sectional time-series study used the TriNetX Network to analyze adults (ages ≥ 18) with chronic, non-infectious uveitis treated with methotrexate, mycophenolate, azathioprine, cyclosporine, tacrolimus, cyclophosphamide, adalimumab, infliximab and/or etanercept. Data was grouped into consecutive 3-year intervals, and Cochran–Armitage tests assessed changes in the proportion of patients treated with each medication.

**Results:**

The total population included 12,035 adult patients. Academic and non-academic institutions shared similar trends in IMT usage. Overall, there was a reduction in the proportion of patients on methotrexate (44.2% in 2005–2007 to 37.6% in 2023–2025, *p* < 0.0001), azathioprine (15.1% in 2005–2007 to 6.7% in 2023–2025, *p* < 0.0001), cyclophosphamide (2.5% in 2011–2013 to 0.8% in 2023–2025, *p* < 0.0001), infliximab (18.6% in 2005–2007 to 14.1% in 2023–2025, *p* < 0.0001) and etanercept (9.0% in 2005–2007 to 2.0% in 2023–2025, *p* < 0.0001). Conversely, treatment with adalimumab rose markedly among patients with chronic uveitis (8.0% in 2005–2007 to 34.6% in 2023–2025; *p* < 0.0001). There was no change in the proportion of patients on mycophenolate (14.6% in 2005–2007 to 12.8% in 2023–2025; *p* = 0.5963).

**Conclusion:**

Key shifts in management of chronic uveitis include the emergence adalimumab as a key steroid sparing agent, the continued role of methotrexate as the first-line steroid-sparing agent, and a marked decline in the use of many older immunomodulatory therapies.

**Supplementary Information:**

The online version contains supplementary material available at 10.1186/s12348-025-00552-z.

## Introduction

Chronic uveitis is defined as intraocular inflammation that persists for more than 3 months or exhibits a relapsing course within 3 months after stopping treatment [1]. The prevalence in the United States is reported at approximately 121 per 100,000 adults, although recent data suggests the true prevalence has been increasing over time [2]. Additionally, it is characterized as a potentially blinding condition, accounting for 10–15% of cases of legal blindness in the United States [2]. Given its increasing prevalence and potential to significantly impair quality of life, appropriate treatment is essential for the effective management of chronic uveitis.

Management often requires immunomodulatory therapy (IMT) to control inflammation while minimizing corticosteroid exposure [3]. IMT refers to a range of systemic immunosuppressive medications that includes conventional non-biologic agents such as antimetabolites (methotrexate, mycophenolate mofetil, azathioprine), calcineurin inhibitors (cyclosporine, tacrolimus), alkylating agents (cyclophosphamide) and biologic therapies such as tumor necrosis factor-alpha (TNF-α) inhibitors (adalimumab, infliximab, etanercept) [3]. In one study, 71% of patients with chronic non-infectious uveitis required systemic IMT to achieve disease quiescence, a state necessary to avoid vision loss in these patients [4]. 

Over the past two decades, the practice patterns of IMT use have evolved in response to accumulating clinical evidence and drug safety profiles [3, 5, 6]. Notably, adalimumab, a fully human anti-TNF-α monoclonal antibody, emerged as an effective therapy [5, 6]. The VISUAL I and II trials demonstrated significant reduction in uveitis flares among patients treated with adalimumab, leading to its 2016 FDA approval as the first on-label drug for the management of non-infectious uveitis [5, 6]. Conversely, etanercept, a soluble TNF-α receptor fusion protein, has shown inferior efficacy in uveitis and has been linked to paradoxical new-onset uveitis in patients treated for systemic inflammatory conditions [7]. Additionally, these biologic agents have demonstrated superior safety profiles compared to older cytotoxic agents like azathioprine and cyclophosphamide, with lower risk of bone marrow suppression and malignancy [8, 9]. Current practice usually reserves cyclophosphamide for refractory, severe uveitis cases [8]. Given these evolving insights, it is important to quantify if these developments have translated into real-world treatment patterns. The current study aims to examine trends in systemic IMT usage among patients with chronic uveitis in the United States over time and compare these findings in both academic and non-academic practice settings.

## Methods

This is a cross-sectional time series study utilizing the TriNetX United States Research Network, a federated network of de-identified electronic health records from various healthcare organizations across the USA. The TriNetX platform allows for the aggregation of clinical data across diverse institutions. This study was exempt from institutional review board (IRB) approval because only de-identified data was analyzed. Further, the study adhered to the principles of the Declaration of Helsinki.

### Study population

All data was collected on July 10th, 2025, from the TriNetX Health Research Network. Diagnoses are represented in TriNetX using the International Classification of Disease, Tenth Revision, Clinical Modification code set (ICD-10) as well as RxNorm, a standardized, normalized naming system for clinical drugs in the US.

As defined by the Standardization of Uveitis Nomenclature (SUN) criteria, adult patients (≥ 18 years of age) with chronic uveitis were defined as those with iridocycitis (H20.X), chorioretinal inflammation (H30.X, which includes intermediate uveitis H30.2) or panuveitis (H44.11) with a repeat instance that occurred at least 3 months after the initial use of the ICD-10 code [10]. All patients with chronic uveitis that started one of the nine IMTs within 1-year of diagnosis were analyzed. It is worth noting that the indication for IMT cannot be definitively established, since some patients may have been prescribed these agents for systemic auto-immune conditions other than uveitis.

**Fig. 1:**
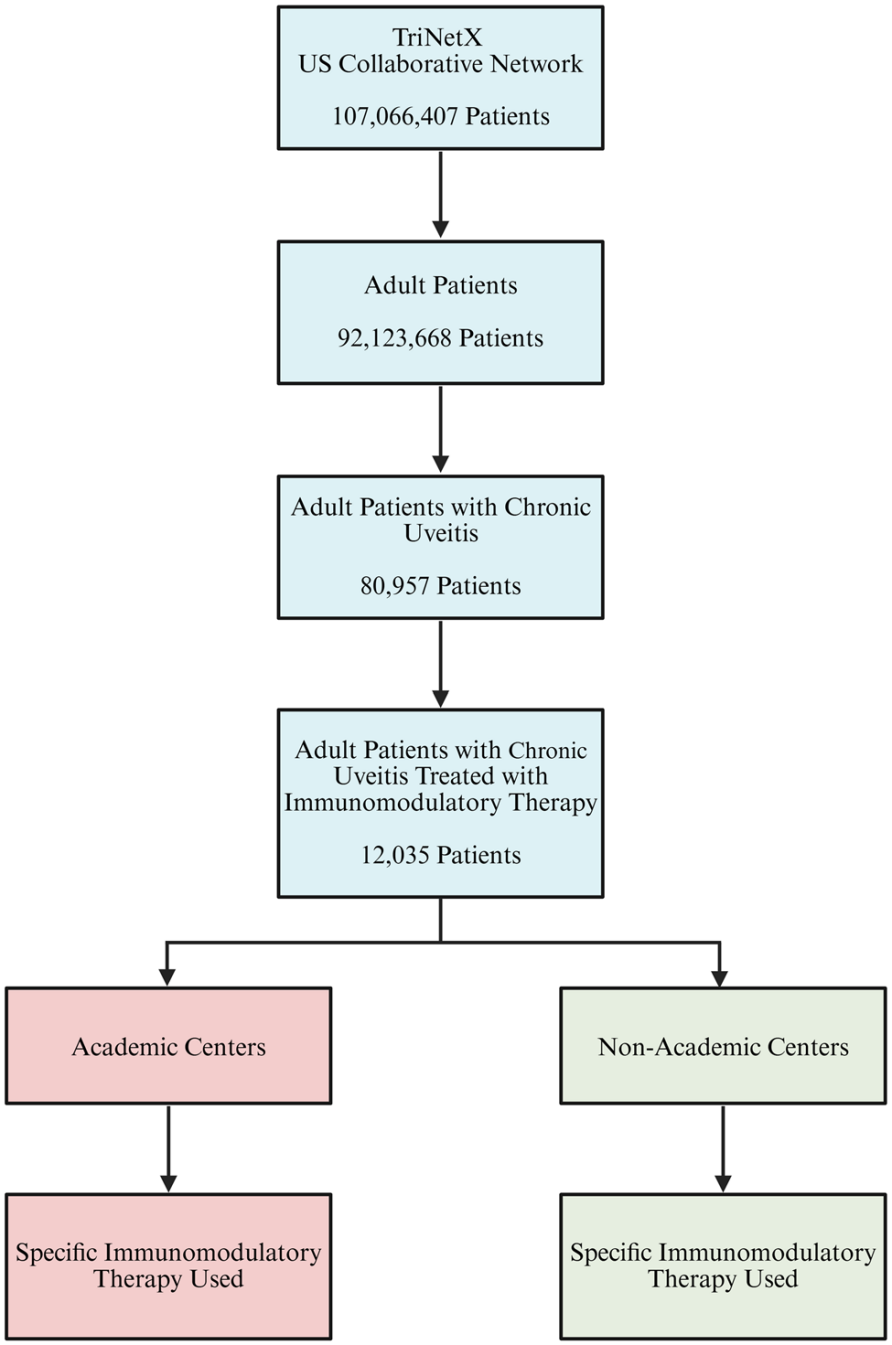
Flowchart illustrating cohort selection and stratification of patients. From the TriNetX US Collaborative Network of 107,066,407 patients, 12,035 adults diagnosed with chronic uveitis and treated with immunomodulatory therapy were analyzed. Created with BioRender.com (accessed August 2025)

The IMTs evaluated included methotrexate (RxNorm: 6851), mycophenolate mofetil (68149, excluding topical formulations)/mycophenolate (265323), azathioprine (1256), tacrolimus (42316, excluding topical, rectal and ophthalmic formulations), cyclosporine (3008, excluding ophthalmic formulations), cyclophosphamide (3002), etanercept (214555), infliximab (191831) and adalimumab (326361). Newer agents such as tocilizumab (612865), secukimumab (1599789), tofacitinib (1357536), baricitinib (2047232), upadacitinib (2196092), basiliximab (196102) as well as the older agents leflunomide (27169) and chlorambucil (2346) were not included as the number of cases were ≤ 10 patients in ≥ 5 (71%) defined time periods. The TriNetX platform does not provide data in these instances to protect patient privacy. To be considered on IMT long term, the patients required a repeat prescription of the medication at least 1 year after the initial instance [5, 6]. Fig. 1 is a flowchart illustrating cohort selection in the current study.

For each patient, information was collected on the systemic IMT used the treatment of chronic uveitis. The dataset was then aggregated by three-year intervals to observe temporal trends (grouped as 2005–2007, 2008–2010, 2011–2013, 2014–2016, 2017–2019, 2020–2022, and 2023–2025). Three-year intervals were selected in the current study because the TriNetX Network does not report data for time periods with ≤ 10 cases to protect patient privacy. Evaluating at shorter intervals led to the omission of data from most time intervals prior to 2015. Within each interval, the number of patients receiving each of the nine IMTs listed above were recorded. Patients receiving more than one IMT concurrently were counted once for each medication received. Patients who met inclusion criteria for chronic uveitis during multiple time intervals were included in each corresponding analysis for which they qualified. Furthermore, patients were categorized into two categories based on the healthcare facility providing care: those treated at registered academic centers and those treated at non-academic centers, as designated within the TriNetX platform. In time intervals where the numbers of cases were ≤ 10 within each subgroup, subtraction was used from the total to obtain the exact number of cases.

### Statistical analysis

The Cochran–Armitage test for trend was used to assess whether the proportion of patients receiving each medication changed significantly across ordered 3-year intervals as previously described [11]. These tests were applied separately for academic and non-academic centers. The relative distribution of IMT usage between academic and non-academic centers in the 2005-2007 and 2023-2025 time intervals was compared to assess convergence or divergence in practice. To adjust for multiple hypothesis testing, Benjamini-Hochberg correction was performed. An adjusted two-tailed test was performed to assess trends in each medication. A p-value < 0.05 was considered statistically significant for trend analyses. All statistical tests were performed using R studio version 2024.04.2 + 764.

## Results

### Baseline characteristics

The total population included 12,035 adult patients with a mean age of 52 ± 19 (range 18–90). Systemic rheumatologic conditions known to be associated with uveitis were identified in 5277 (43.8%) patients. However, due to the limitations inherent to the TriNetX database, the temporal or causal relationship between these systemic diagnoses and uveitis could not be definitively established. Females comprised 64.1% of patients. Of the study population, 61.1% identified as White, 20.5% as African American/ Black 9.45% as Hispanic/ Latino. A total of 10,945 (91%) patients had a previous diagnosis of iridocyclitis, 5,714 (47%) had a previous diagnosis of chorioretinal inflammation and 1,758 (15%) had a previous diagnosis of posterior cyclitis. These diagnostic categories were not mutually exclusive; patients could have one or more of these diagnoses. However, each patient was counted only once in the overall analysis. A total of 11,877 (98.6%) patients had been previously treated with glucocorticoids at one point in time, 7,067 (58.7%) with methotrexate, 3,929 (32.6%) with mycophenolate, 1,681 (14.0%) with azathioprine, 1,317 (10.9%) with systemic cyclosporin, 1,120 (9.3%) with systemic tacrolimus, 452 (3.8%) with cyclophosphamide, 5,961 (49.5%) with adalimumab, 2,214 (18.4%) with infliximab and 981 (8.2%) with etanercept.

### Trends over time in academic institutions

Immunomodulatory therapy used in the treatment of chronic uveitis in adults seen at US academic institutions can be seen in Table 1. The proportion of chronic uveitis patients on each specific IMT was also calculated as a proportion of the total patients on IMT and plotted over time (Supplemental Fig. 1). Trends included a reduction in the proportion of patients on methotrexate (46.7% in 2005–2007 to 37.3% in 2023–2025, *p* < 0.0001), azathioprine (16.2% in 2005–2007 to 5.4% in 2023–2025, *p* < 0.0001), cyclophosphamide (1.3% in 2011–2013 to 0.5% in 2023–2025, *p* = 0.0082) and etanercept (10.2% in 2005–2007 to 1.7% in 2023–2025, *p* < 0.0001). There was no change in the proportion of patients on mycophenolate (13.7% in 2005–2007 to 14.2% in 2023–2025, *p* = 0.9117). Conversely, there was an increase in the proportion of patients on adalimumab (9.6% in 2005–2007 to 35.7% in 2023–2025, *p* < 0.0001).

**Table 1:** Patients treated with immunomodulatory therapy for chronic uveitis from academic centers in the United States were included. Cochran-Armitage trend tests were performed to assess trends in medication prescriptions over time

Medication	2005–2007	2008–2010	2011–2013	2014–2016	2017–2019	2020–2022	2023–2025	*P*-value
Methotrexate	78 (46.7%)	137 (40.3%)	526 (41.8%)	1055 (41.1%)	1538 (39.6%)	1785 (37.5%)	1578 (37.3%)	**< 0.0001**
Mycophenolate	23 (13.8%)	48 (14.1%)	168 (13.3%)	390 (15.2%)	655 (16.9%)	735 (15.5%)	601 (14.2%)	0.9117
Azathioprine	27 (16.2%)	44 (12.9%)	104 (8.3%)	189 (7.4%)	263 (6.8%)	296 (6.2%)	228 (5.4%)	**< 0.0001**
Cyclosporine	NA	NA	13 (1.0%)	34 (1.3%)	44 (1.1%)	40 (0.8%)	26 (0.6%)	**0.0048**
Tacrolimus	NA	NA	22 (1.7%)	63 (2.5%)	107 (2.8%)	149 (3.1%)	113 (2.7%)	0.0799
Cyclophosphamide	NA	NA	16 (1.3%)	32 (1.2%)	52 (1.3%)	45 (0.9%)	23 (0.5%)	**0.0082**
Adalimumab	16 (9.6%)	44 (12.9%)	162 (12.9%)	476 (18.6%)	1040 (26.8%)	1600 (33.6%)	1511 (35.7%)	**< 0.0001**
Infliximab	28 (16.8%)	52 (15.3%)	222 (17.6%)	426 (16.6%)	550 (14.2%)	603 (12.7%)	581 (13.7%)	**< 0.0001**
Etanercept	17 (10.2%)	28 (8.2%)	58 (4.6%)	91 (3.5%)	112 (2.9%)	118 (2.5%)	73 (1.7%)	**< 0.0001**
Total Patients	167	340	1259	2566	3880	4756	4232	NA

### Trends over time in non-academic institutions

Immunomodulatory therapy used in the treatment of chronic uveitis in adults seen at non-academic centers in the US can be seen in Table 2. The proportion of chronic uveitis patients on each specific IMT was also calculated as a proportion of the total patients on IMT and plotted over time (Supplemental Fig. 2). Similarly, there was reduction in the proportion of patients on methotrexate (51.0% in 2008–2010 to 37.6% in 2023–2025, *p* = 0.0028) and infliximab (29.4% in 2008–2010 to 15.8% in 2023–2025, *p* = 0.0004), but an increase in the proportion of patients on adalimumab (11.8% in 2008–2010 to 29.1% in 2023–2025, *p* < 0.0001).

**Table 2:** Patients treated with immunomodulatory therapy for chronic uveitis from non-academic centers in the United States were included. Cochran-Armitage trend tests were performed to assess trends in medication prescriptions over time

Medication	2005–2007	2008–2010	2011–2013	2014–2016	2017–2019	2020–2022	2023–2025	*P*-value
Methotrexate	15 (46.9%)	52 (51.0%)	148 (44.2%)	246 (41.6%)	307 (36.5%)	370 (37.8%)	322 (37.6%)	**0.0028**
Mycophenolate	6 (18.8%)	11 (10.8%)	25 (7.5%)	50 (8.5%)	66 (7.8%)	79 (8.1%)	50 (5.8%)	**0.0269**
Azathioprine	3 (9.4%)	14 (13.7%)	77 (23.0%)	95 (16.1%)	105 (12.5%)	106 (10.8%)	115 (13.4%)	**0.0011**
Cyclosporine	NA	NA	5 (1.5%)	16 (2.7%)	31 (3.7%)	16 (1.6%)	16 (1.9%)	0.3617
Tacrolimus	NA	NA	1 (0.3%)	12 (2.0%)	29 (3.4%)	25 (2.6%)	15 (1.8%)	0.4566
Cyclophosphamide	NA	NA	23 (6.9%)	19 (3.2%)	22 (2.6%)	24 (2.5%)	18 (2.1%)	**0.0007**
Adalimumab	0 (0%)	12 (11.8%)	46 (13.7%)	122 (20.6%)	217 (25.8%)	238 (24.3%)	249 (29.1%)	**< 0.0001**
Infliximab	9 (28.1%)	30 (29.4%)	72 (21.5%)	117 (19.8%)	156 (18.5%)	169 (17.3%)	135 (15.8%)	**0.0004**
Etanercept	1 (3.1%)	3 (2.9%)	18 (5.4%)	24 (4.1%)	30 (3.6%)	36 (3.7%)	28 (3.3%)	0.3401
Total Patients	32	102	335	591	842	978	857	NA

### Trends over time in all institutions

**Fig. 2:**
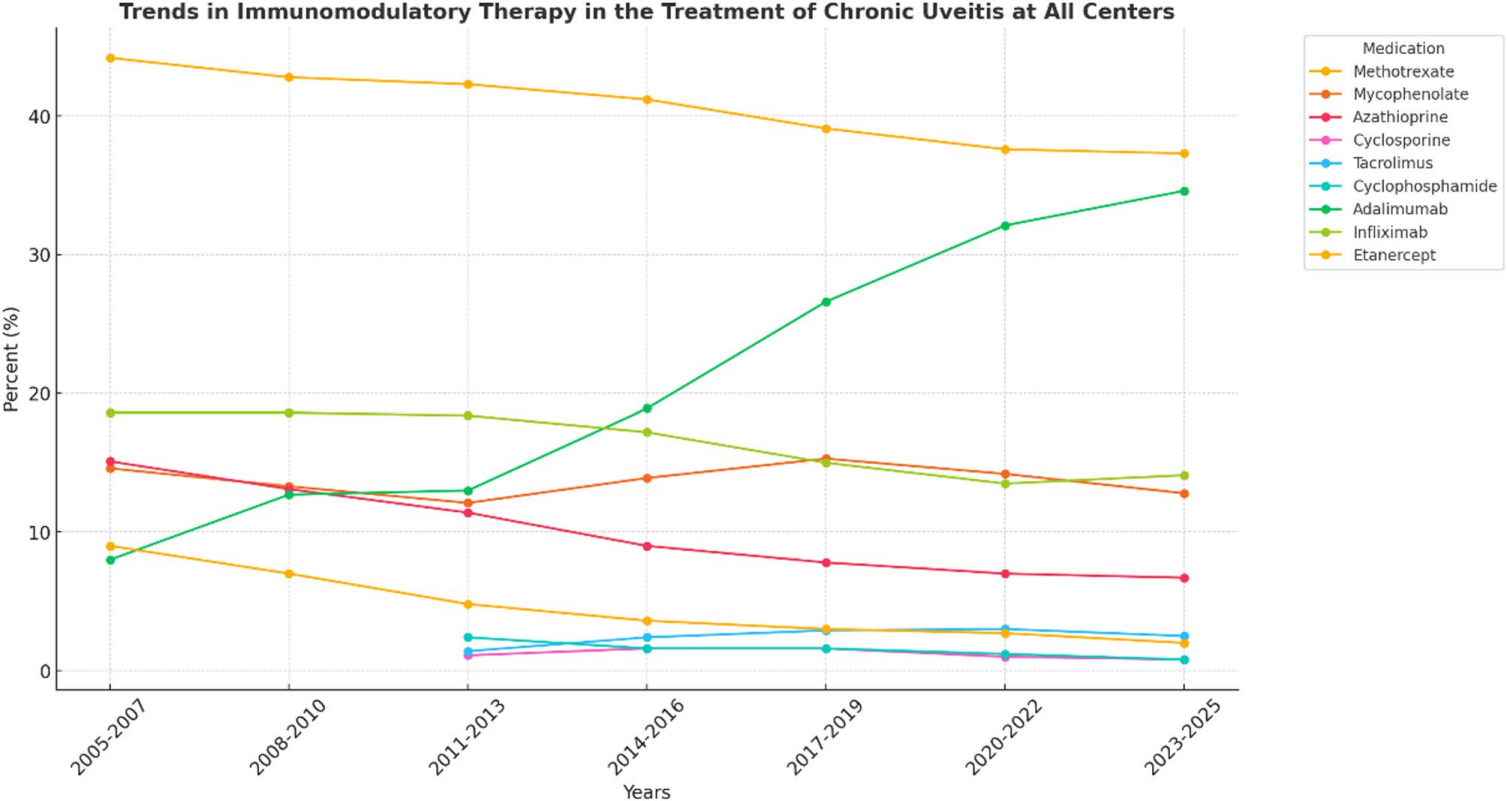
Line graph illustrating trends in immunomodulatory therapy in the treatment of chronic uveitis at all US centers between 2005 and 2025. ChatGPT-40 was utilized on August 3rd, 2025 to create this figure

Immunomodulatory therapy used in the treatment of chronic uveitis in all adults in the US can be seen in Table 3. The proportion of chronic uveitis patients on each specific IMT was also calculated as a proportion of the total patients on IMT and plotted over time (Fig. 2). Trends in included a reduction in the proportion of patients on methotrexate (44.2% in 2005–2007 to 37.6% in 2023–2025, *p* < 0.0001), azathioprine (15.1% in 2005–2007 to 6.7% in 2023–2025, *p* < 0.0001), cyclophosphamide (2.5% in 2011–2013 to 0.8% in 2023–2025, *p* < 0.0001), infliximab (18.6% in 2005–2007 to 14.1% in 2023–2025, *p* < 0.0001) and etanercept (9.0% in 2005–2007 to 2.0% in 2023–2025, *p* < 0.0001). There was no change in the proportion of patients on mycophenolate (14.6% in 2005–2007 to 12.8% in 2023–2025, *p* = 0.5963). Conversely, there was an increase in the proportion of patients treated with adalimumab (8.0% in 2005–2007 to 34.6% in 2023–2025, *p* < 0.0001).

**Table 3:** Patients treated with immunomodulatory therapy for chronic uveitis from both academic and non-academic centers in the United States were included. Cochran-Armitage trend tests were performed to assess trends in medication prescriptions over time

Medication	2005–2007	2008–2010	2011–2013	2014–2016	2017–2019	2020–2022	2023–2025	*P* value
Methotrexate	88 (44.2%)	189 (42.8%)	674 (42.3%)	1301 (41.2%)	1845 (39.1%)	2155 (37.6%)	1900 (37.3%)	**< 0.0001**
Mycophenolate	29 (14.6%)	59 (13.3%)	193 (12.1%)	440 (13.9%)	721 (15.3%)	814 (14.2%)	651 (12.8%)	0.5963
Azathioprine	30 (15.1%)	58 (13.1%)	181 (11.4%)	284 (9.0%)	368 (7.8%)	402 (7.0%)	343 (6.7%)	**< 0.0001**
Cyclosporine	NA	NA	18 (1.1%)	50 (1.6%)	75 (1.6%)	56 (1.0%)	42 (0.8%)	**0.0023**
Tacrolimus	NA	NA	23 (1.4%)	75 (2.4%)	136 (2.9%)	174 (3.0%)	128 (2.5%)	0.0526
Cyclophosphamide	NA	NA	39 (2.4%)	51 (1.6%)	74 (1.6%)	69 (1.2%)	41 (0.8%)	**< 0.0001**
Adalimumab	16 (8.0%)	56 (12.7%)	208 (13.0%)	598 (18.9%)	1257 (26.6%)	1838 (32.1%)	1760 (34.6%)	**< 0.0001**
Infliximab	37 (18.6%)	82 (18.6%)	294 (18.4%)	543 (17.2%)	706 (15.0%)	772 (13.5%)	716 (14.1%)	**< 0.0001**
Etanercept	18 (9.0%)	31 (7.0%)	76 (4.8%)	115 (3.6%)	142 (3.0%)	154 (2.7%)	101 (2.0%)	**< 0.0001**
Total Patients	199	442	1594	3157	4722	5734	5089	NA

## Discussion

The current study examines trends in IMT in patients with chronic uveitis from 2005 to 2025. One prior study described real-world IMT prescribing patterns among 158 uveitis specialists, with treatments administered to Medicare beneficiaries, predominantly patients aged 65 or older [12]. The current study differs in that it analyzes patterns of prescription regardless of insurance-type in all adult patients, and explores differences between academic and non-academic centers. Among Medicare beneficiaries, the most commonly prescribed medication was methotrexate, similar to the results seen in the current study [12]. However, adalimumab only accounted for 18.5% of IMT prescriptions and was less common than mycophenolate, which accounted for 28% of prescriptions [12]. The current study found adalimumab to comprise 32.1% of IMTs during the similar time period indicating that there may be age or insurance specific factors that could change practice patterns. While the current study supports the prior findings in a broader patient population, it also highlights some differences, indicating that other variables may contribute to variations in treatment trends.

The most important finding in the current study is the widespread adoption of adalimumab, which transitioned from an off-label option in the mid-2000s to becoming the second most commonly used IMT in recent years when considering all adults with uveitis. Notably, it is the only FDA-approved systemic therapy for non-infectious uveitis, which likely improved access and contributed to the observed increase in prescriptions. However, adalimumab was approved by the FDA in 2016, but the current study shows the increase in adalimumab use began even before 2016. This early increase was likely driven by emerging evidence in the 2000s that TNF-α blocking antibodies could achieve similar efficacy with a better safety profile compared to older IMT agents [13, 14]. A recent randomized clinical trial found that in cases of severe systemic Behcet’s disease, induction therapy with infliximab led to a superior complete response rate compared to cyclophosphamide [15]. In the current study, adalimumab use accelerated further after FDA approval and eventually surpassed the use of infliximab. While no comparative trials have evaluated these two biologics specifically in uveitis, they are generally considered similarly effective for ocular inflammation. The preference for adalimumab between 2016 and 2025 may therefore reflect practical advantages such as subcutaneous self-administration, lower infusion burden and higher insurance approval rates rather than differences in efficacy. By the early 2020s, the use of adalimumab plateaued, which likely represents a saturation of its target population as it became the standard of care. It may also reflect the recent introduction of adalimumab biosimilars and other biologics that target IL-6 or IL-17 which could diversity uveitis therapy going forward [16, 17]. 

In contrast with the rise of adalimumab, the downward trajectory of etanercept underscores that not all TNF-α inhibitors are equally effective for uveitis. Both clinical experience and randomized trials have shown that etanercept is ineffective in controlling ocular inflammation, with some cases reporting paradoxical onset or exacerbation of uveitis [18]. Consequently, etanercept usage dwindled in the current study, falling to 2.0% by 2023–2025 which aligns with guidelines that now explicitly caution against using etanercept for uveitis.

The traditional steroid-sparing antimetabolite IMTs, methotrexate, mycophenolate and azathioprine have long formed the backbone of treatment for chronic uveitis. However, the current study indicates that the prescription patterns have diverged over time. Methotrexate, in particular, remains the most commonly prescribed IMT at all time periods, although its use has declined over the years. The preference for methotrexate may reflect its well-recognized role in managing systemic rheumatologic diseases associated with uveitis, where it effectively addresses both ocular and systemic inflammation [19]. In contrast, mycophenolate may be less effective at controlling systemic inflammation [20]. However, unlike the management of systemic symptoms, high-level evidence suggests that methotrexate and mycophenolate have comparable efficacy in the treatment of uveitis. A randomized clinical trial in non-infectious uveitis found no statistically significant difference in inflammation control between methotrexate and mycophenolate, although the results favored methotrexate in a non-statistically significant manner [21]. Despite this, in real-world practice, methotrexate was used 2–3 times more often than mycophenolate for the treatment of uveitis. Several potential reasons may explain this with one major factor being the cost as studies have found methotrexate to be significantly less expensive than mycophenolate in the United States [21, 22]. In fact, mycophenolate is estimated to have a 5-fold higher cost for comparable dosing [21, 22]. Another factor is tolerability; when methotrexate is co-administered with folic acid supplementation, many patients experience minimal side effects with only 9% reporting gastrointestinal upset in one study [23]. In contrast, mycophenolate use is associated with gastrointestinal side effects in up to 21.2% of patients [24]. Moreover, methotrexate’s role in rheumatology has bolstered confidence in its use for uveitis [25]. It is often combined with biologics in rheumatologic disease to enhance efficacy, and it is well documented that concomitant methotrexate use can reduce anti-drug antibody formation against TNF-α inhibitors like adalimumab [26]. Given the trend of increasing TNF-α inhibitor use, this synergistic effect has allowed methotrexate use to remain high.

In contrast, azathioprine use declined significantly over the 20-year period. Azathioprine was one of the earliest IMTs used in ocular inflammation and has substantial literature to support its use [27, 28]. However, the current study demonstrated that by the 2020s, azathioprine accounted for a small fraction of IMT prescriptions, especially in academic institutions. Several reasons may explain this trend. Firstly, azathioprine has a less favorable safety profile compared to methotrexate and mycophenolate. In a large cohort study, approximately 25% of patients had to discontinue azathioprine within the first-year of treatment due to toxicities [27]. Despite this tradeoff, unlike stronger agents like cyclophosphamide, there is a paucity of evidence comparing azathioprine to methotrexate or mycophenolate. Thus, many specialists likely prefer agents such as adalimumab over azathioprine, given the superior efficacy and tolerability.

Meanwhile, the use of the alkylating agent cyclophosphamide was found to decrease with time. In the early 2000s, cyclophosphamide was often used for cases of sight-threatening uveitis such as Behcet’s disease, serpiginous choroiditis or necrotizing scleritis [29, 30]. The current study shows that by the 2020s, cyclophosphamide is rarely used in both academic and nonacademic settings. One possible explanation is the growing body of evidence showing that biologics can often eliminate the need for cyclophosphamide. As mentioned above, infliximab was demonstrated to have superiority compared to cyclophosphamide in the setting of severe Behcet’s disease [15]. Likewise, other case series have reported high success using anti-TNF-α agents in uveitis that previously required cyclophosphamide [31, 32]. 

Finally, our findings suggest that overall practice patterns in IMT were more similar than different between academic and non-academic settings from 2005 to 2025. Both types of centers showed the same general trends discussed above. The notable differences we did observe were relatively subtle: for instance, mycophenolate use remained stable in academic centers, whereas it declined in community practice; conversely, azathioprine was phased out in academics but saw a bit more prolonged use in the community. These divergences are difficult to conclusively explain, but we can speculate on a few contributing factors. One factor may be physician familiarity and comfort level with certain drugs. If certain providers in the community trained when azathioprine was the primary option and have more experience with this medication, they may have continued to prescribe it for their patients as long as the uveitis remained controlled and the patients tolerated the drug [33]. In addition, the types of cases seen may have also influenced prescribing patterns [33, 34]. Finally, system-level factors like formulary preferences or insurance approvals can differ between large academic institutions and smaller practices, potentially influencing prescription patterns [35]. 

This study has some limitations worth noting. First, we utilized a claims database, isolating patients with chronic uveitis treated with IMT by requiring an immunosuppressive prescription after the uveitis diagnosis. However, we cannot be certain that each IMT was prescribed specifically for chronic uveitis. Some patients may have been prescribed these medications for a parallel systemic condition. Therefore, trends for agents with lower prescription counts should be interpreted with caution [36]. We attempted to mitigate this limitation by focusing on relative changes over time, but the possibility of confounding by indication remains. Second, the current study relies on the accuracy of diagnostic and billing codes, so any errors in medical coding could lead to misclassification and affect our analysis [37]. Lastly, due to TriNetX Network privacy constraints, which suppress data for cohorts with ten or fewer cases, we were unable to analyze IMT trends for individual uveitis subtypes (such as anterior or posterior uveitis). As a result, potential subtype-specific variations in treatment utilization may not have been fully captured in this analysis.

## Conclusions

In conclusion, the current study provides a comprehensive overview of how the management of chronic uveitis in the United States has evolved over the last 20 years. The key shifts include the rise of biologic use, with adalimumab in particular rising to an established standard of care, methotrexate remaining as the first-line steroids-sparing agent and reduction in the use of older IMTs such as azathioprine, etanercept and cyclophosphamide. These trends are not surprising given the robust prospective randomized clinical trial data supporting the use of adalimumab. Ongoing research and surveillance will be essential to optimize IMT use, particularly as emerging therapies such as anti-interleukin agents and tyrosine kinase inhibitors begin to enter the uveitis treatment landscape.

## Supplementary Information

Below is the link to the electronic supplementary material.

Supplemental Figure 1. Line graph illustrating the trends in immunomodulatory therapy in the treatment of chronic uveitis at US academic institutions between 2005 and 2025. ChatGPT-40 was utilized on August 3rd 2025 to create this figure.

Supplemental Figure 2. Line graph illustrating trends in immunomodulatory therapy in the treatment of chronic uveitis at US non-academic centers between 2005 and 2025. ChatGPT-40 was utilized on August 3rd, 2025 to create this figure.

## Data Availability

No datasets were generated or analysed during the current study.
