# Supplementary figures and images for: Trends in immunomodulatory therapy for the treatment of chronic uveitis in the United States
Source: J Ophthalmic Inflamm Infect. 2025 Nov 25;15:100. doi: 10.1186/s12348-025-00552-z (PMC12748333; doi:10.1186/s12348-025-00552-z)

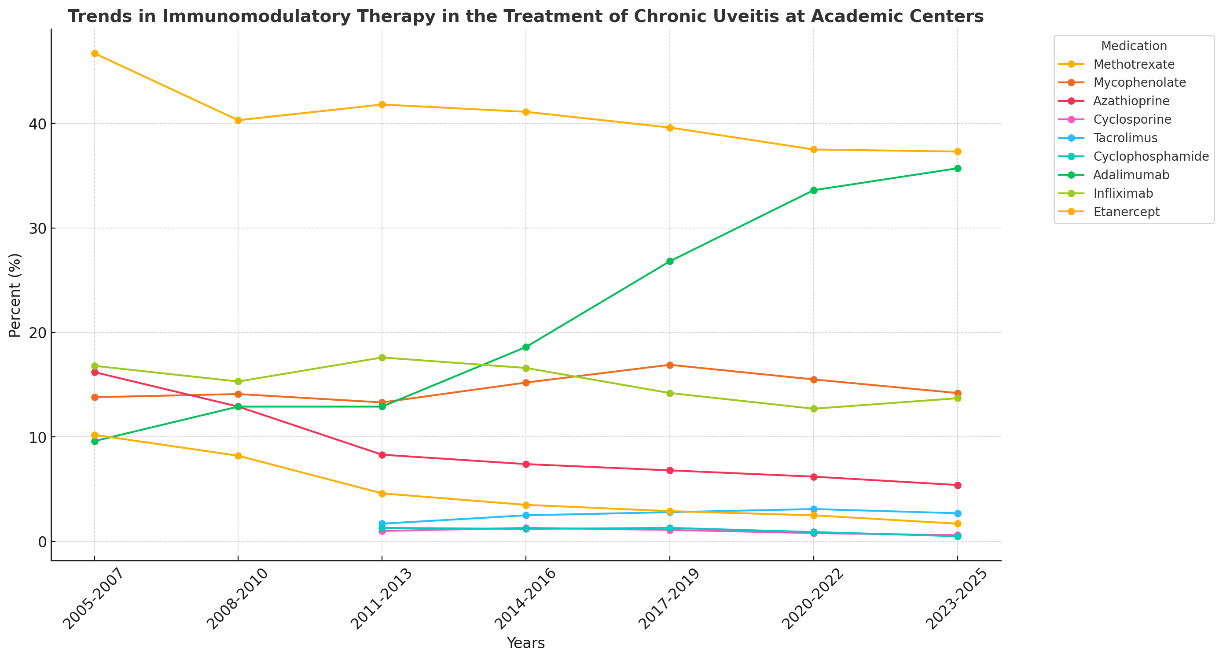

Supplement: Supplementary file 1 — Supplemental Figure 1. Line graph illustrating the trends in immunomodulatory therapy in the treatment of chronic uveitis at US academic institutions between 2005 and 2025. ChatGPT-40 was utilized on August 3rd 2025 to create this figure. [file 12348_2025_552_MOESM1_ESM.png]

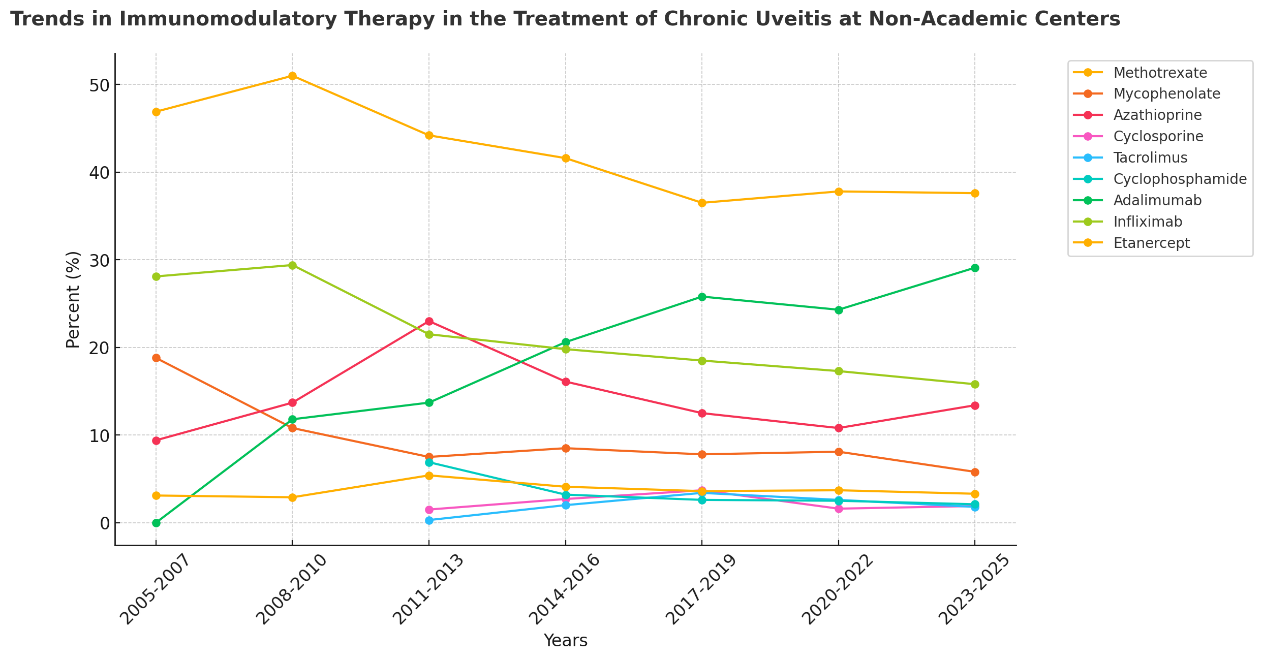

Supplement: Supplementary file 2 — Supplemental Figure 2. Line graph illustrating trends in immunomodulatory therapy in the treatment of chronic uveitis at US non-academic centers between 2005 and 2025. ChatGPT-40 was utilized on August 3rd, 2025 to create this figure. [file 12348_2025_552_MOESM2_ESM.png]
